# Solar-Blind Photodetectors for Harsh Electronics

**DOI:** 10.1038/srep02628

**Published:** 2013-09-11

**Authors:** Dung-Sheng Tsai, Wei-Cheng Lien, Der-Hsien Lien, Kuan-Ming Chen, Meng-Lin Tsai, Debbie G. Senesky, Yueh-Chung Yu, Albert P. Pisano, Jr-Hau He

**Affiliations:** 1 Institute of Photonics and Optoelectronics & Department of Electrical Engineering, National Taiwan University, Taipei 10617, Taiwan, ROC; 2 Applied Science and Technology Program & Department of Electrical Engineering and Computer Science, University of California, Berkeley, CA 94720, USA; 3 Institute of Physics, Academia Sinica, Taipei 11529, Taiwan, ROC; 4 Department of Aeronautics & Astronautics, Stanford University, Stanford, CA 94305, USA; 5 Department of Electrical and Computer Engineering, University of California, San Diego, CA 92093, USA; 6 These authors contributed equally to this work.

## Abstract

We demonstrate solar-blind photodetectors (PDs) by employing AlN thin films on Si(100) substrates with excellent temperature tolerance and radiation hardness. Even at a bias higher than 200 V the AlN PDs on Si show a dark current as low as ~ 1 nA. The working temperature is up to 300°C and the radiation tolerance is up to 10^13^ cm^−2^ of 2-MeV proton fluences for AlN metal-semiconductor-metal (MSM) PDs. Moreover, the AlN PDs show a photoresponse time as fast as ~ 110 ms (the rise time) and ~ 80 ms (the fall time) at 5 V bias. The results demonstrate that AlN MSM PDs hold high potential in next-generation deep ultraviolet PDs for use in harsh environments.

Solar-blind deep ultraviolet (DUV) photodetectors (PDs) with excellent thermal stability and reliability have attracted a strong interest owing to their broad potential applications in the fields of solar observations, ultraviolet (UV) astronomy, military defense, automatization, short-range communications security, as well as environmental and biological researches^1^,^2^. It is very important that as considering the constrains for above-mentioned practical applications, the operation of photodetection systems in harsh environments is required inevitably^3^. For example, for the next envisaged space missions solar-blind DUV PDs capable of operating at high temperatures and radiation are a crucial ingredient for studying the Sun (*e.g.*, the Solar Orbiter)^4^.

Solar-blind DUV PDs fabricated from wide-bandgap materials, such as BN^5^, Al_x_Ga_1−x_N^2^,^6^, diamond^7^, LaAlO_3_^8^ and In_2_Ge_2_O_7_^9^,^10^ with intrinsic solar-blindness and low dark currents would not need Wood's filters to eliminate the longer wavelength and heavy cooling systems to reduce the dark current as conventional Si-based PDs and commercial photomultiplier tubes do^11^,^12^,^13^,^14^,^15^,^16^. In addition, wide-bandgap materials are suitable for high-temperature and high-power applications due to their high thermal conductivity and breakdown field strength in comparison to Si (1.5 W/cm°C and 0.3 MV/cm, respectively), the most widely used semiconductor material for the PDs^16^,^17^. Moreover, most of the fabrication processes for the wide-bandgap materials are complex and costly as well as not easy to integrate with peripheral Si-based electronics^18^. For example, typically AlN was grown on SiC or sapphire substrates using metal organic chemical vapor deposition^6^. Up to date, few reports were discussed on the general suitability of above-mentioned materials for extremely harsh environments although they have been proved their absorption ability at the DUV region (190–350 nm)^5^,^6^,^7^. Clearly, solar-blind DUV PDs for extremely harsh environments are still at their early stage of development.

AlN with direct bandgap (~6.2 eV), high thermal conductivity (3.2 W/cm°C), high breakdown field (4–6 MV/cm), excellent thermal stability as well as radiation hardness is an ideal candidate for the development of solar-blind DUV PDs in harsh environment applications^19^,^20^,^21^,^22^. So far, conventional AlN-based epitaxial layers were grown either on sapphire or on SiC substrates, leading to the high substrate cost, which is an obstacle for developing AlN electronics. Recently, it is reported that AlN thin films can be successfully grown on Si substrates, offering the advantages of reduced cost and large-area manufacture^23^,^24^,^25^.

In this study, we demonstrate solar-blind Schottky PDs with back-to-back MSM geometry by employing AlN thin films on Si(100) substrates using the reactive sputtering deposition for use in harsh environments. The AlN MSM PDs on Si show the low dark current of ~ 1 nA and no sign of breakdown even at a bias higher than 200 V. Excellent thermal stability and radiation hardness of solar-blind AlN MSM PDs are achieved. The working temperature is up to 300°C and the radiation tolerance is up to 10^13^ cm^−2^ of 2-MeV proton fluences for AlN MSM PDs. As compared with the characteristic parameters of the pre-existing devices (order of seconds), the AlN MSM PDs show a faster photoresponse (~110 ms of the rise time and ~ 80 ms of the fall time) at 5 V bias. The results demonstrate the high promise of AlN as an active material for solar-blind DUV photodetection in extremely harsh environments.

## Results

### Fabrication and characterization of AlN PDs

**Figure 1:**
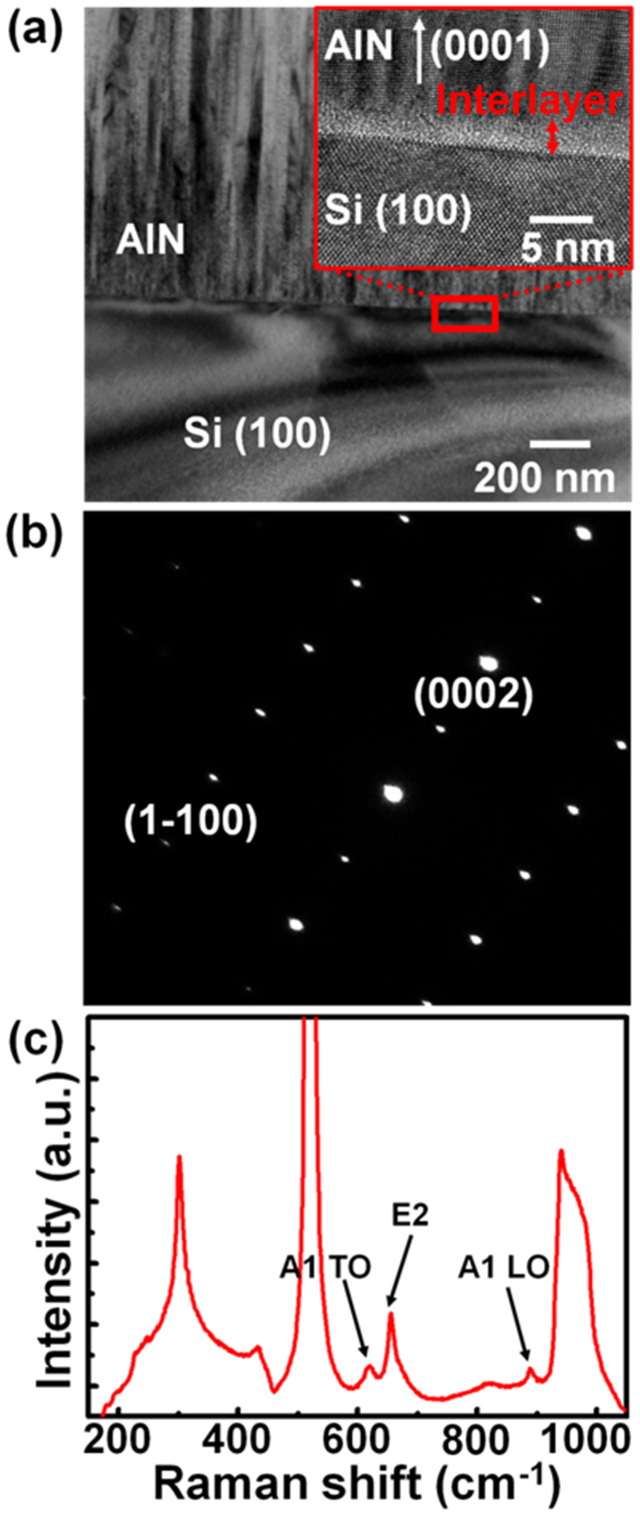
(a) Cross-sectional TEM image of the AlN thin films deposited on a Si(100) substrate. The inset shows the high-resolution TEM image of the marked area in (a). (b) The electron-diffraction pattern of the AlN films. (c) Raman spectrum of a 1-μm-thick AlN on Si(100) deposited by reactive sputtering at 350°C.

Figure 1a shows the cross-sectional transmission electron microscopy (TEM) images of the AlN/Si(100) layered structure, revealing that the AlN films consist of oriented columnar grains that are perpendicular to the (100) surface of the Si substrates. A clear thin interface with a thickness of ~ 3 nm is observed, as shown in high-resolution TEM image in the inset of Figure 1a. The interface layers are highly defective and disordered due to the large lattice mismatch between Si substrates and AlN layers^24^. The electron diffraction pattern with an aperture size of 150 nm, as shown in Figure 1b, indicates that AlN layers are highly texture crystals which are superior to those reported previously^26^. The Raman peaks associated with the AlN layers are shown in Figure 1c, *i.e.*, A1 symmetry transverse optical (TO) mode at 618.5 cm^−1^, planar E2 symmetry high mode at 656.9 cm^−1^ and axial A1 symmetry longitudinal mode (LO) at 888.4 cm^−1^. This indicates that AlN layers with a hexagonal crystal structure have been successfully deposited on the Si(100) substrates, echoing the TEM observations^24^.

**Figure 2:**
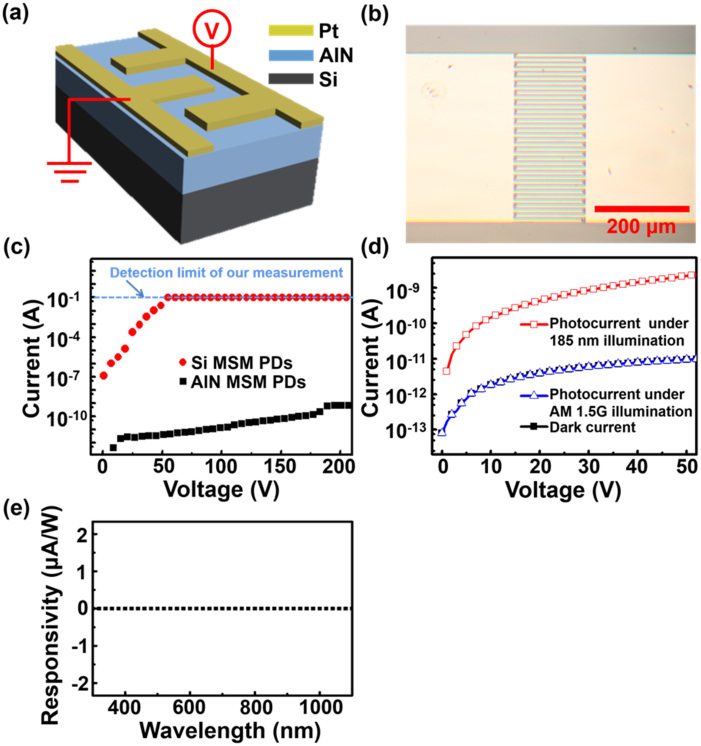
(a) Schematic and (b) optical microscopic image of the AlN MSM PDs. (c) *I*–*V* curves of AlN and Si MSM PDs in the dark. Note that the 0.1 A is the detection limit of the electrical measurements. (d) *I*–*V* curves of the AlN MSM PDs measured in the dark and under AM 1.5G illumination and 185-nm light illumination. (e) The wavelength-dependent responsivity of the AlN PDs measured under 5 V bias.

A schematic and an optical microscopic image of the AlN MSM PDs are depicted in Figure 2. In order to boost the photosensitivity of AlN layers, the devices were fabricated in MSM geometry with Schottky contacts to AlN to effectively lower the dark current^27^. Accordingly, 200-nm-thick Pt layers as Schottky contacts with 20-nm-thick Ti adhesion layers were deposited on AlN layers using sputtering deposition. Figure 2c shows the current–voltage (*I*–*V*) curves of AlN MSM PDs measured in the dark. To distinguish the excellent characteristics of AlN MSM PDs, we also compare Si MSM PDs here. The AlN MSM PDs exhibit a dark current as low as ~ 1 nA and no sign of breakdown at a bias up to 200 V (Note that 200 V is the measurement limit of our system). In contrast, the Si MSM PDs show the electric breakdown at a bias of ~ 50 V. These superior characteristics are directly attributed to the outstanding material properties of AlN, such as large energy bandgap, high breakdown electric field and excellent mechanical strength. To demonstrate the solar-blind DUV photosensitivity of the AlN MSM PDs, the *I*–*V* characteristics of the AlN MSM PDs were measured in the dark and under air mass 1.5 global (AM 1.5G) illumination and 185-nm light illumination, as shown in Figure 2d. The sensitivity factor of a PD, photo-to-dark current ratio (PDCR) is defined as (*I*_ph_−*I*_d_)/*I*_d_, where *I*_ph_ is the photocurrent and *I*_d_ is the dark current^28^. At 5 V bias, the PDCR values of AlN MSM PDs are 0 and 63 under AM 1.5G and 185-nm light illumination, respectively, suggesting that there is no solar light absorption in AlN films because the bandgap of AlN is larger than the energy (4.42 eV) of the shortest wavelength (280 nm) in AM 1.5G solar spectrum. Moreover, as shown in Figure 2e, the wavelength-dependent responsivity of the AlN PDs is zero from 300 nm to 1100 nm, again confirming its solar-blind characteristics.

### Photodetction of AlN devices in harsh environments

**Figure 3:**
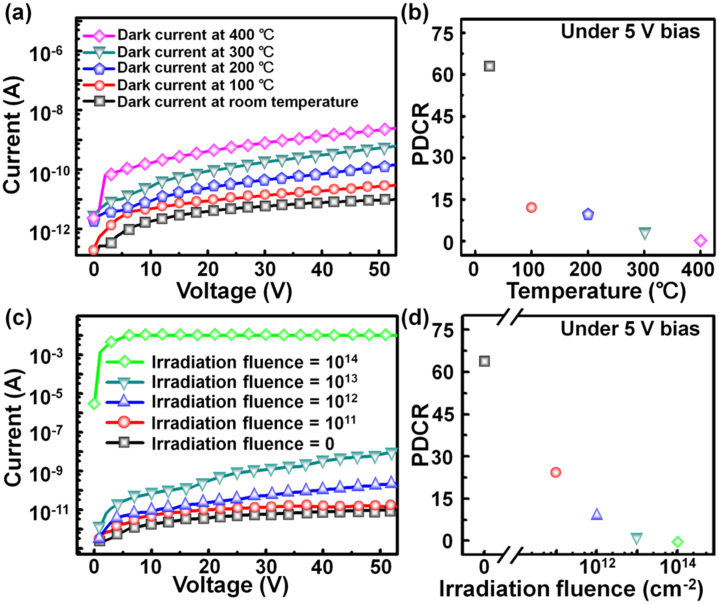
(a) *I*–*V* curves of the AlN MSM PDs measured in the dark at different working temperatures. (b) PDCR value as function of temperature under 5 V bias and 185-nm light illumination (*I*_light_ = 100 mW/m^2^). (c) *I*–*V* curves of the AlN MSM PDs as a function of fluence of 2-MeV proton irradiation measured in the dark at room temperature. (d) PDCR value as function of 2-MeV proton irradiation fluence under the bias of 5 V and 185-nm light illumination (*I*_light_ = 100 mW/m^2^) at room temperature.

To demonstrate photodetection applications under the high-temperature environments, the thermal stability of the AlN MSM PDs is evaluated by measuring the *I*–*V* in the dark under different temperature conditions. As shown in Figure 3a, the dark current is increased as the temperature increases because of the thermal-generation current based on the relation exp(-*E_g_*/2*kT*), where *E_g_* is the bandgap of AlN, *T* is operation temperature and *k* is the Boltzmann constant^29^. The responsivity of the AlN PDs can be also estimated to be ~ 0.015 A/W at room temperature under 5 V bais and 185-nm light illumination by *R* = *I*_p_/*P*, where *R* is the responsivity, *I*_p_ is the photocurrent, and *P* is the illumination power^30^. It should be noted that the responsivity of AlN MSM PDs on Si could be further enhanced by increasing applied bias and reducing contact spacing. The external quantum efficiency of AlN PDs is further estimated to be up to ~ 10% (assuming the photogain = 1) at 185-nm wavelength by *R = η_ext_Gq/hν*, where *η_ext_* is the external quantum efficiency, *G* is the photogain, *q* is the electronic charge, *h* is Planck's constant and ν is the frequency of the incident wavelength^11^. The temperature-dependent PDCR under 185-nm illumination and a 5 V bias in Figure 3b indicates that the AlN MSM PDs are capable of photodetection up to 300°C (the PDCR value of 3.5), mainly due to small levels of leakage current and high thermal stability of AlN at high temperatures. A further increase in temperature lowers the PDCR of AlN MSM PDs due to the raise of dark current which cannot be completely eliminated at higher temperatures^31^. When the temperature increases to 400°C, the PDCR value decreases to zero, indicating that the AlN MSM PDs cannot work properly. After the working temperature is decreased to the temperature lower than 400°C, AlN MSM PDs are fully recovered, showing the reversibility of fabricated AlN PDs. We note that as compared to very low dark current (~0.6 pA under the bias of 5 V at room temperature), the high thermal noise of the probe tips used in the characterization setup should be considered when the AlN MSM PDs are characterized at high temperature measurements. Therefore, to further boost the operation temperature for the AlN MSM PDs, the thermal noise generated from the probe of characterization setups must be reduced by using the suitable high-temperature ceramic packages or a high-temperature probe station^32^,^33^. Furthermore, we envision that further improvements such as the passivation layers for the surface defect reduction or the reinforcement of Pt/AlN interface might be able to extend the working temperatures of the AlN MSM PDs^34^,^35^.

In order to highlight the high radiation tolerance for space applications, the AlN MSM PDs were irradiated at room temperature with the 2-MeV proton fluences ranging from 10^11^ cm^−2^ to 10^14^ cm^−2^. Note that the protons with the energy less than 2 MeV occupy a volume of earth's space with the fluences ranging from 10^1^ cm^−2^ to 10^8^ cm^−2^ (the corresponding region at geocentric distances of about 1*L*–12*L*, where *L* is approximately equal to the geocentric distance of a field line in the geomagnetic equator)^36^. Figure 3c shows the *I*–*V* curves of AlN MSM PDs after proton irradiation measured in the dark at room temperature. The increase in dark current with proton fluence is due to the proton-induced displacement damage^37^. To quantitatively evaluate the tendency of the proton irradiation fluences and photosensing characteristics of the AlN MSM PDs, the PDCR versus the proton irradiation fluence is measured as shown in Figure 3d. It shows that the AlN MSM PDs are capable of photodetection even after proton irradiation exposure with the fluences of 10^13^ cm^−2^, suggesting that the AlN MSM PDs are well suited for DUV detection in the space environment due to very high radiation hardness of AlN. As a proton irradiation fluence is increased to 10^14^ cm^−2^, the PDCR value decreases to zero, indicating that the dark current increases largely with the huge generation of proton-induced displacement damage at high irradiation fluences, leading to the degradation of AlN MSM PDs.

### Fast photoresponse of AlN PDs

**Figure 4:**
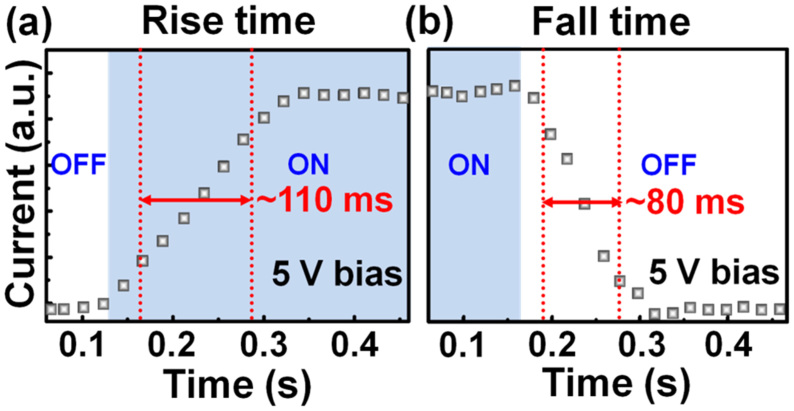
The transient photocurrent of AlN MSM PDs measured at room temperature under 5 V bias and 185-nm light illumination (*I*_light_ = 100 mW/m^2^).

The operation speed of the MSM PDs can be determined by performing the time-resolved measurement. Figure 4 presents the photocurrent as a function of time under 185-nm light illumination (light intensity *I*_light_ = 100 mW/m^2^) under a fixed bias of 5 V. A fully reversible response is acquired; under illumination, the current rises to a high value and returns to a low value when the light is on (ON state) and off (OFF state), respectively. The transition between ON and OFF states reveals the response/recovery speed of the AlN MSM PDs. The results are shown in Figure 4, from which the rise time (from 10% to 90% of the maximum photocurrent as switching light from OFF to ON) and the fall time (from 90% to 10% of the maximum photocurrent as switching light from ON to OFF) of the AlN MSM PDs can be estimated to be 110 and 80 ms, respectively. We expect that the photoresponse times of AlN MSM PDs will be further improved *via* reducing surface defects and metal contact spacing^38^,^39^.

## Discussion

In summary, the MSM Schottky AlN PDs on Si substrates show a dark current as low as ~ 1 nA at a bias up to 200 V. Operating at the temperatures up to 300°C with the PDCR value of 3.5 and tolerating 2-MeV proton irradiation at the fluences up to 10^13^ cm^−2^ with the PDCR value of 0.7 show the excellent temperature and radiation hardness of fabricated AlN MSM PDs. In addition, the temporal response of photocurrent of the AlN MSM PDs reveals the response times and recovery times as fast as ~ 110 ms and ~ 80 ms, respectively. The excellent optical properties of AlN promise a new generation of stable, fast, solar-blind DUV PDs for the extremely harsh electronic applications, such as sensing, imaging, and intrachip optical interconnects in high temperature and high radiation environments.

## Methods

### AlN film growth and characterization

Highly textured 1-μm-thick AlN films were grown on p-type Si(100) with a resistivity of 1–50 Ωcm fabricated at approximately 350°C using a reactive sputtering of a pure Al (99.999%) target with a combination of nitrogen and argon as the sputtering gasses. This sputtering system used a dual ac target technology (40 kHz–10 kW power supply) which mitigates the disappearing anode problems generally experienced when using stander dc or radio frequency sputtering tools^23^. After the growth process, film crystallinity quality of the resulting products was probed by Raman spectroscope (HORIBA Jobin Yvon LabRAM) with a He-Ne laser excitation wavelength of 633 nm. Field-emission transmission electron microscopy (JEOL JEM-2100F, operated at 200 kV) was used to investigate the microstructures of AlN films.

### AlN MSM PD fabrication and characterization

The MSM PDs were defined using photolithography with active areas of 500 × 158 μm^2^, and utilized 8-μm-wide, 150-μm-long and 200-nm-thick interdigitated Pt electrodes with 8-μm-wide spacing to serve as Schottky contacts on AlN/Si substrates for the MSM PDs, as shown in Figure 2. After fabrication process of devices, A low-pressure mercury lamp was employed to act as the 185-nm light source to characterize AlN MSM PDs. The wavelength-dependent responsivity measurements were carried out using the EQE-R3011 spectral response system (Enli Technology Co., Ltd.) equipped with the Xenon lamp as light source. Moreover, for radiation tolerance testing, AlN MSM PDs were irradiated at room temperature using a 2 MeV proton beam from a 3 MV tandem accelerator (NEC 9SDH-2, National Electrostatics Corporation). The typical current of the proton beam was 2–50 nA (the current increases with increasing fluences), with the beam fluences ranged from 10^11^ cm^−2^ to 10^14^ cm^−2^ at the sample target. The Keithley 4200-SCS semiconductor characterization system was used to measure *I–V* characteristics of the fabricated AlN MSM PDs. For high-temperature testing, the AlN MSM PDs are heated on a hot plate, and the device temperature was monitored with a calibrated thermocouple (K type).

## Author Contributions

D.S.T. and J.H.H. conceived the experiment. AlN films were prepared by W.C.L., D.G.S. and A.P.P. D.S.T. and W.C.L. fabricated the AlN MSM PDs. D.H.L. and M.L.T. measured the operation speed of the AlN MSM PDs. K.M.C. and Y.C.Y. tested the radiation tolerance of AlN MSM PDs. D.S.T. and J.H.H. wrote the manuscript.
